# Supplementary material for: Exome Sequencing Identifies Genetic Variants Associated with Extreme Manifestations of the Cardiovascular Phenotype in Marfan Syndrome
Source: Genes (Basel). 2022 Jun 8;13(6):1027. doi: 10.3390/genes13061027 (PMC9223058; doi:10.3390/genes13061027)
Supplement: Supplementary file 1 [file genes-13-01027-s001.zip › supplementary materials.pdf]

Supplemental Material

Exome sequencing identifies genetic variants associated with extreme manifestations of the cardiovascular phenotype in Marfan Syndrome

Yanireth Jimenez [1], Cesar Paulsen [2], Eduardo Turner [2] , Sebastian Iturra [2] , Oscar Cuevas [2,3] , Guillermo Lay-son [4], Gabriela Repetto [5], Marcelo Rojas [1] and Juan Calderon [6,7,*]

Familial or sporadic patients	Patient ID code	Mild Phenotype			Severe Phenotype			Classification	Sex (M/F)	Age of onset of cardivascular surgery (years)
		AoR* diameter with Z ≤2 in adults, no major cardiovascular event	AoR diameter with Z ≤1 in pediatric patients, no major cardiovasc ular event	Age at first major cardiovascular event ≥30 years old	AoR diameter with Z ≥3 in adults	AoR diameter with Z ≥2 in pediatric patients	One or more major cardiovascular event before 18 years old			
Family 1	CAS-01-001				X			Severe	F	-
	CAS-01-002	X						Mild	M	-
	CAS-01-003	X						Mild	M	-
	CAS-01-004							Unaffected	F	-
	CAS-01-005				X			Severe	M	-
	CAS-01-006							Unaffected	M	-
	CAS-01-007	X						Mild	F	-
Family 2	CAS-01-019			X				Mild	F	31
	CAS-01-020		X					Mild	F	-
	CAS-01-021		X					Mild	F	-

	CAS-01-022		X					Mild	F	-
	CAS-01-023			X				Mild	F	32
**Family 3**	CAS-01-024			X				Mild	F	37
	CAS-01-025							Unaffected	M	-
	CAS-01-026					X		Severe	M	-
**Family 4**	CAS-01-035	X						Mild	M	-
	CAS-01-036		X					Mild	M	-
**Family 5**	CAS-01-046			X				Mild	M	30
	CAS-01-048		X					Mild	M	-
	CAS-01-045						X	Severe	M	16
**Non-familial cases**	CAS-01-016					X		Severe	M	-
**Non-familial cases**	CAS-01-027				X			Severe	M	27
**Non-familial cases**	CAS-01-031						X	Severe	M	12
**Non-familial cases**	CAS-01-043						X	Severe	M	16

**Non-familial cases**	CAS-01-044	X						Mild	F	-

Table S1. Criteria for the stratification and classification of patients with MFS according to the severity of the aortic phenotype. Mild and severe phenotypes were defined based on deviation from the criteria published on the Revised Ghent Nosology for MFS (patients who are at the extremes of the curve)

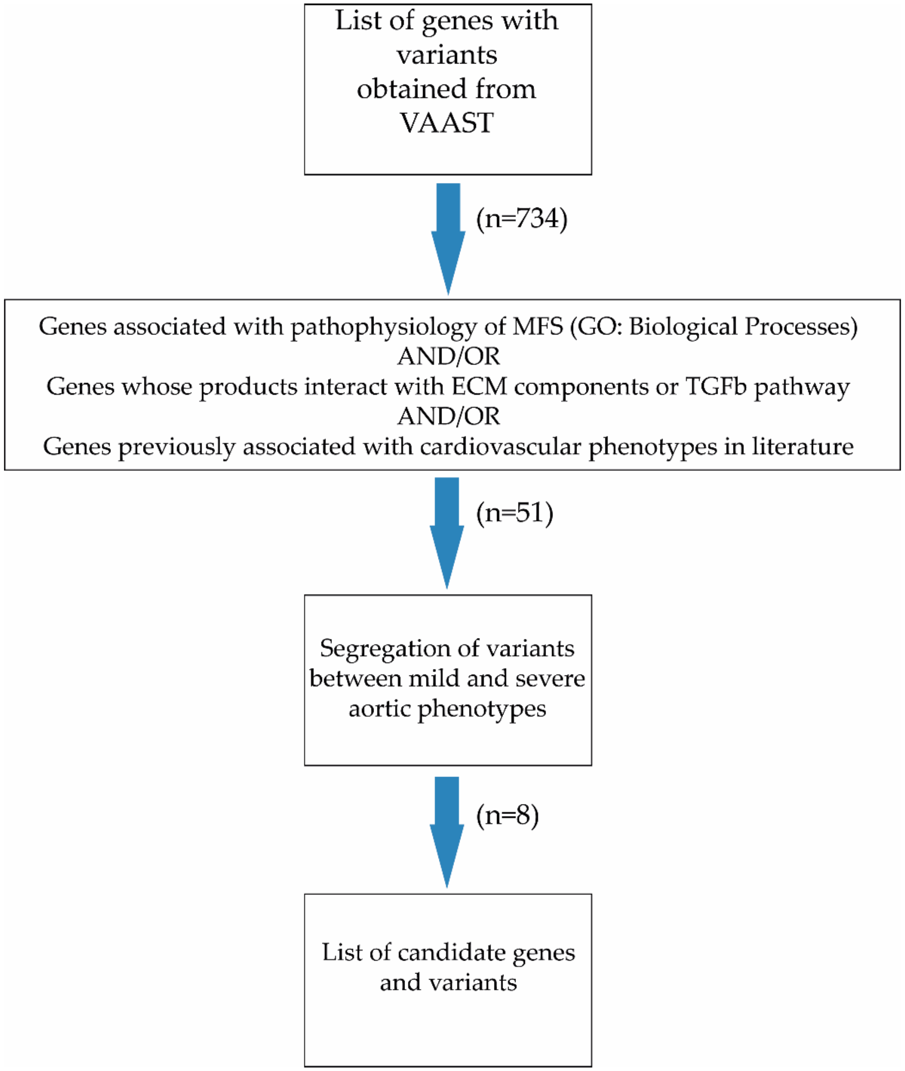

Figure S1. Flowchart of genes and variant prioritization to select candidate modifiers of the aortic phenotype in MFS

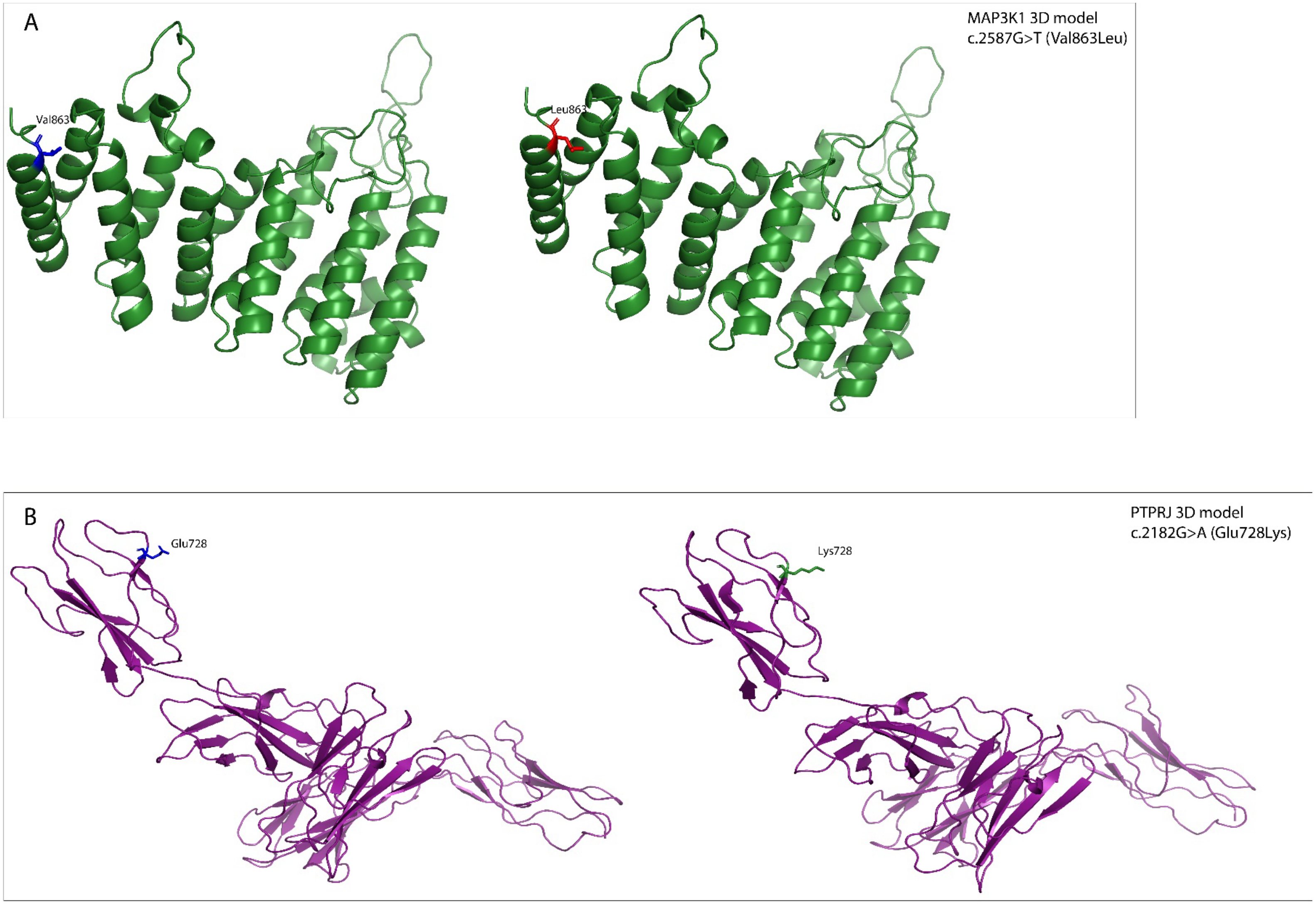
A
MAP3K1 3D model
c.2587G>T (Val863Leu)
Val863
Leu863
B
PTPRJ 3D model
c.2182G>A (Glu728Lys)
Glu728
Lys728

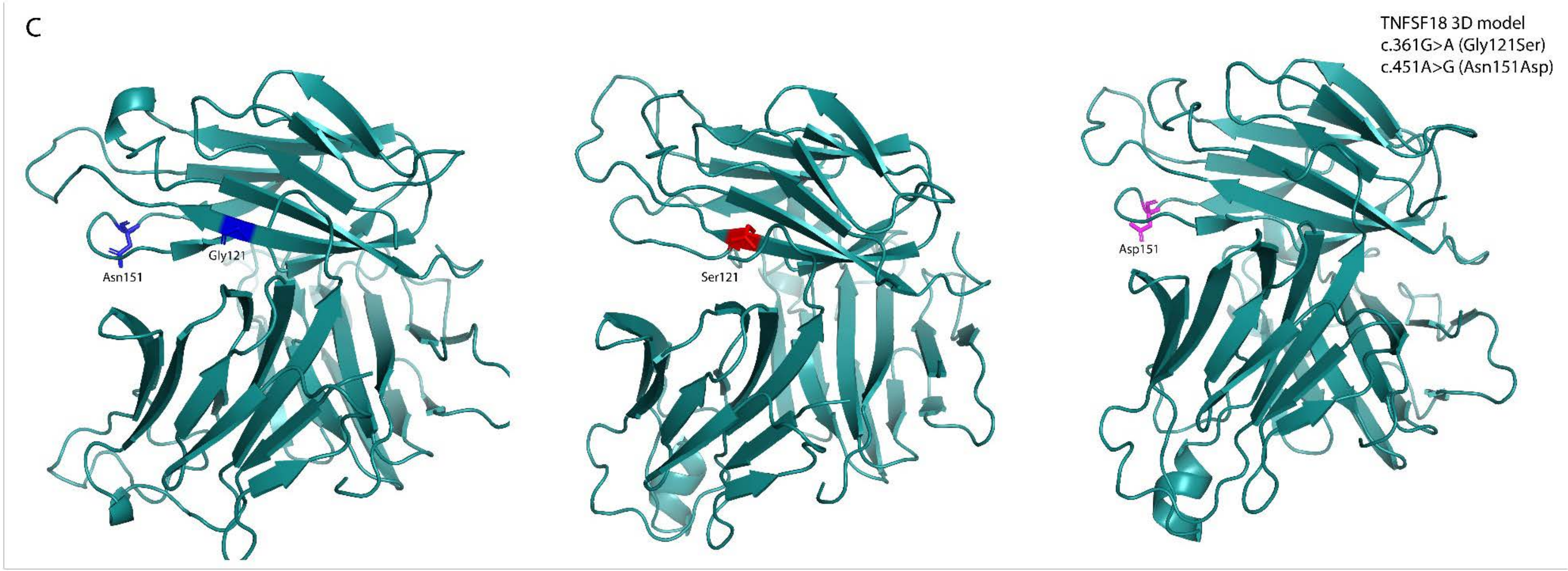
C
TNFSF18 3D model
c.361G>A (Gly121Ser)
c.451A>G (Asn151Asp)
Asn151
Gly121
Ser121
Asp151

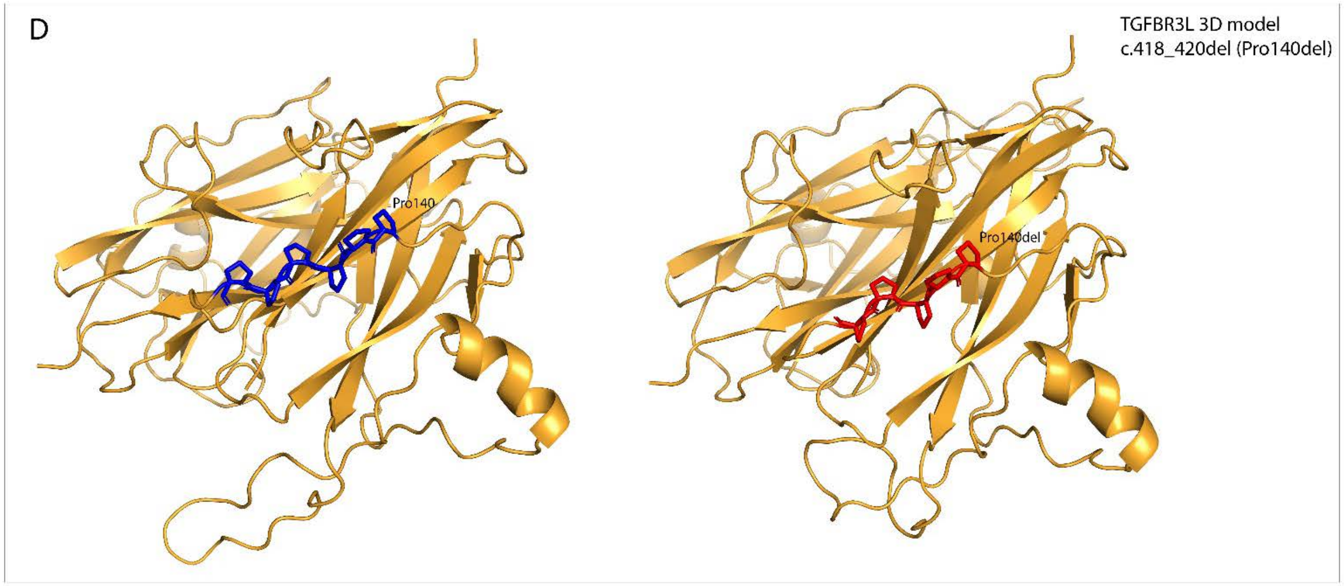
D
TGFBR3L 3D model
c.418_420del (Pro140del)
Pro140
Pro140del

FigureS2. 3D structural modeling of MAP3K1, PRPRJ, TNFSF18 AND TGFBR3L proteins to show the predicted effect of the variants identified in MFS patients with extreme presentations of the aortic phenotype. Structures of both reference and alternative residues are displayed.

(A) Left panel shows the reference structure of MAP3K1. Right panel shows that variant c.2587G>T causes a change of the amino acid valine for the amino acid leucine at position 863 (Val863Leu). **(B)** Left panel shows the reference structure of PTPRJ. Right panel shows that variant c.2182G>A produces a change of Glu to Lys at position 728 (Glu728Lys). **(C)** Left panel shows the reference structure of TNFSF18. Middle panel shows that variant c.361G>A produces a change of Gly to Ser at position 121 (Gly121Ser). Right panel shows that variant c.451A>G produces a change of Asn to Asp at position 151 (Asn151Asp). **(D)** Left panel shows the reference structure of TGFBR3L. Right panel shows that variant c418_420del causes a loss of the amino acid proline at position 140 (Pro140del).
